# Usefulness of postoperative surveillance MR for women after breast-conservation therapy: Focusing on MR features of early and late recurrent breast cancer

**DOI:** 10.1371/journal.pone.0252476

**Published:** 2021-06-11

**Authors:** Jeongmin Lee, Bong Joo Kang, Sung Hun Kim

**Affiliations:** Departments of Radiology, Seoul St. Mary’s Hospital, College of Medicine, The Catholic University of Korea, Seoul, Korea; Medical University of Vienna, AUSTRIA

## Abstract

**Purpose:**

To investigate the imaging characteristics of early and late recurrent breast cancer and the detectability of mammography, ultrasonography, and breast magnetic resonance imaging (MRI) in patients who underwent breast-conservation therapy (BCT).

**Materials and methods:**

Total of 1312 women with 2026 surveillance breast MRI after BCT between January 2014 and September 2018 were studied. Early recurrence was defined as newly diagnosed breast cancer and/or axillary metastasis within 12 months of surgery. Late recurrence was defined as recurrence after 12months of surgery. We assessed the detectability of recurrent lesions in each postoperative imaging modality and evaluated characteristics of recurrent lesions on postoperative MRI by comparing early and late recurrence groups.

**Result:**

Of the 2026 cases, 103 were confirmed as recurrent breast cancer by biopsy or surgery. Thirty-one cases were early recurrence, and 72 cases were late recurrence. MRI showed significantly higher detectability for recurrent lesions (102 cases, 99%) than mammography (59.4%, *p* < 0.001) or ultrasound (68.9%, *p* < 0.001), or both mammography and ultrasound (81.6%, *p* < 0.001). The recurrent lesions did not have typical malignant morphologic features, but variable features on MRI. However, early recurrent lesions showed fast enhancement in early dynamic phase regardless of the kinetic pattern of delayed dynamic phase; and late recurrence lesions showed early fast enhancement and delayed washout pattern. There were 19 cases which were not detected on mammography or ultrasound but could only be detected with MRI.

**Conclusion:**

Postoperative breast MRI showed significantly higher detectability for recurrent lesions than mammography and ultrasound. Early fast enhancement is the most important feature of recurrent lesions on postoperative breast MRI for both early and late recurrence groups. Due to its high possibility of recurrence, further work-up should be considered regardless of their morphologic features.

## Introduction

Over the past decade, breast cancer survival has significantly improved with the development of postoperative treatment. In fact, the 5-year survival rate of breast cancer in Korea has increased from 83.2% to 91.3% [1]. However, women who have been treated for primary breast cancer still have chance in developing a second breast cancer including loco-regional recurrences and contralateral breast cancers [2–4]. Local recurrence can increase the risk of distant metastasis or breast cancer-related death compared to cases without recurrence [5]. Previous studies have shown that early detection of second breast cancers can improve the prognosis of patients with recurrent cancer [6, 7]. For these reasons, postoperative surveillance of breast cancer patients is important and should be done systemically.

Regular mammography alone has been recommended as the postoperative screening tool for patients who underwent breast conservation therapy (BCT) by American Society for Clinical Oncology (ASCO) and National Comprehensive Cancer Network (NCCN). Both guidelines have not recommended breast magnetic resonance imaging (MRI) as a routine postoperative surveillance tool [8, 9]. However, the number of breast MRI for postoperative surveillance has markedly increased recently due to the limitation of mammography for evaluating post-BCT breast [10]. In Korea, the National Health Insurance provides a financial support for cancer patients and the cost for postoperative breast MRI. Based on these backgrounds, the number of postoperative breast MRI has been increasing in Korea.

Despite the increase in number of postoperative breast MRI, there are few studies on imaging characteristics of recurrent breast cancer on MRI. Moreover, there have been no studies to the usefulness of postoperative MRIs according to the time of recurrence of disease in our knowledge. Thus, we investigated the usefulness of postoperative breast MRI focusing on the time interval of cancer recurrence and compared the detectability and imaging characteristics of recurrent lesion on MRI with conventional imaging modalities including mammography and ultrasound at the time of recurrence.

## Materials and methods

### Subjective

This retrospective study was approved by the institutional review board (IRB) of our institution and informed consent was waived by Ethics committee due to its retrospective design. All procedures performed in studies involving human participants were in accordance with the ethical standards of IRB of our institution; and assessments were carried out as per rules of the Declaration of Helsinki of 1975, revised in 2013.

A total of 2026 postoperative breast MRIs performed after BCT between January 2014 and March 2019 were reviewed. Cases confirmed to be tumor recurrence through biopsy or surgery were included. "Recurrence" was defined as either of the followings: 1. recurrent malignancy in ipsilateral and/or contralateral breast; 2. metastasis in axillary lymph node. However, malignancies that are not of breast origin such as phyllodes tumor and melanoma were excluded. Cases of borderline or high-risk lesions such as atypical ductal hyperplasia (ADH) or lobular carcinoma in situ (LCIS) were also excluded.

**Fig 1:**
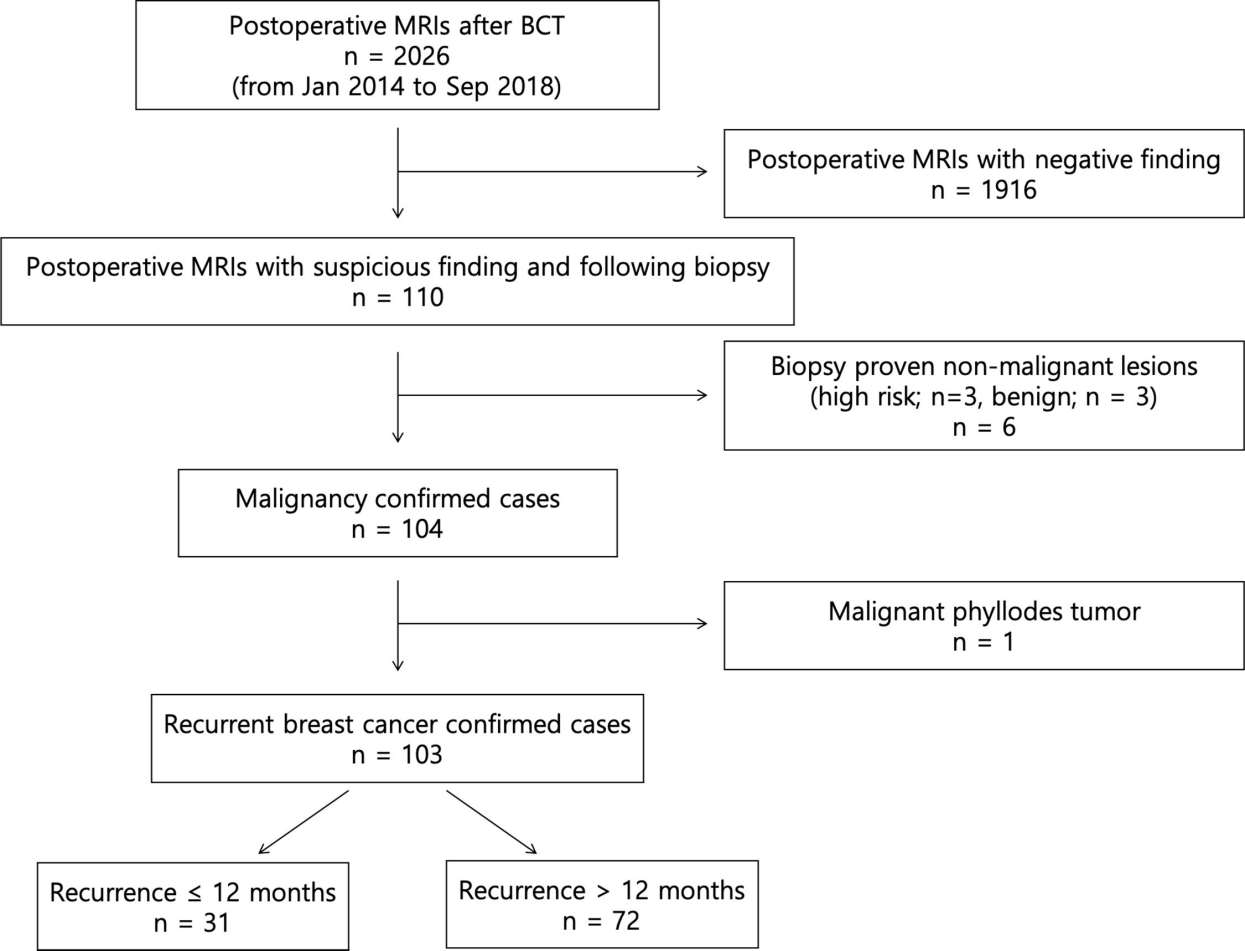
Inclusion and exclusion criteria for patient selection.

Enrolled cases were divided into “early recurrence” group and “late recurrence” group by the time interval between breast surgery and the diagnosis of cancer recurrence. If the time interval between breast surgery and cancer recurrence was 12 months and less, it was defined as “early recurrence.” If the time interval was more than 12 months, it was defined as “late recurrence” (Fig 1).

### Clinical-pathologic information

We reviewed all medical records of the enrolled cases. Recurrence interval was defined as the interval months between the date of first surgery for primary cancer and the date of postoperative MRI where recurrent lesion was detected. The stage of primary cancer by AJCC 7th edition, histology, and the subtype by hormone receptor of primary and recurrent cancer were reviewed.

Ultrasound-guided biopsy or stereotactic biopsy was recommended and performed for all detected suspicious lesions on mammography, ultrasound and MRI. Most of the suspicious lesions detected only on MRI occurred in the breast after BCT; thus, MR-guided biopsy was expected to fail due to architectural distortions. In case of suspicious lesions in the opposite breast without changes after BCT, MR-guided biopsy was impossible due to small size of breast or non-visualization after compression. In cases of the lesions detected only on MRI, a second-look ultrasound was performed by expert breast radiologists. There was no case that could not be found on second look ultrasound.

### MR protocols

The postoperative MR imaging examination was performed with a routine protocol of our institution, which was the same as the pretreatment MRI for initial cancer staging. The patient underwent MRI in prone position using a dedicated bilateral breast surface coil on 3-T MR imaging system. The protocol for MR image acquisition is similar to that of a previous study at our institute [11]. Images were obtained with the following protocol using a 3-T system (Verio; Siemens Healthcare, Erlangen, Germany) (Table 1). T1 dynamic images were obtained after injection of gadopentetate dimeglumine (0.1 mmol/kg, Gadovist; Bayer Schering Pharma, Berlin, Germany).

**Table 1:** MRI protocol.

Sequence	TR/TE (msec)	Flip angle (degrees)	Slice thickness (mm)	FOV (mm^2^)	Matrix	Scan time
**T2-TSE**	3530/93	80	4	350 x 350	576 x 403	2 min 28sec
**T1 Dynamic**	5.0/1.6	12	1.2	350 x 350	544 x 381	1 min

### Imaging analysis

All enrolled cases have corresponding mammography (Lorad Selenia; Hologic, Danbury, USA) and breast ultrasound (iU22; Philips Healthcare, Bothell, USA, Aplio i700; Canon Medical Systems, Tochigi, Japan) images taken at the same time point as breast MRI. Initially, breast ultrasound was performed by radiologists with variable experiences in breast ultrasound. Two expert radiologists with 9 and 20 years of breast imaging reviewed breast MRI, mammography, and breast ultrasound at the time of diagnosis of cancer recurrence. The experts reviewed each image, and evaluated and compared the detectability of each imaging modality. Detectability of mammography, breast ultrasound and breast MRI were compared between early and late recurrent groups; and detectability of combination of three imaging modalities–mammography with MRI, mammography with ultrasound–was also assessed. Not only the detectability was assessed, but also the imaging characteristics of the detected lesions in all imaging modalities were analyzed. Imaging characteristics of suspicious lesions were classified as suspicious or non-suspicious finding according to BI-RADS 5th edition. Consensus was made by two experts in case of discordance in categorization. The MR-only detected lesions found by experts in second look ultrasound were not included in the detectability of ultrasound.

### Statistical analysis

Chi-square test or Fisher’s exact test was used for comparison of characteristics between early and late recurrence groups. Chi-square test was used for comparison of the proportions observed in early and late recurrence groups.

## Results

### Subjects

One hundred and three cases of breast cancer recurrence were finally included. All enrolled patients underwent preoperative or pretreatment breast MRI after initial breast cancer diagnosis. In addition, most of the patients underwent first postoperative breast MRI within 12 months after BCT. Of the 103 cases, 31 cases were classified as early recurrence group and the other 72 cases were classified as late recurrence group. There were 10 cases that omitted radiation therapy and 8 cases that omitted any of the postoperative treatment including radiation therapy, hormone therapy, chemotherapy or target therapy. Except these 18 cases, all recurrent cases were treated with standard therapy for post-BCT state. Also, every case had negative resection margin in pathologic reports.

The median age of cancer diagnosis was 48 years (41 to 56) and median age of cancer recurrence was 51 years (44 to 59). There was no significant difference in the median age of cancer diagnosis between early recurrence group and late recurrence group. However, the median age of cancer recurrence in early recurrence group was significantly younger than the late recurrence group (*p* = 0.038). Luminal B subtype was the most common subtype, and triple negative type was the second most common subtype in both primary and recurrent cancer. Other characteristics of the enrolled subjects are summarized in Table 2.

**Table 2:** Characteristics of patients.

	Total	Early recurrence	Late recurrence	
	N = 103	N = 31	N = 72	*p* value
**Age**				
Cancer diagnosis				0.523
Median (IQR)	48 (41, 56)	47 (41, 56)	48 (41.5, 56)	
Cancer recurrence				0.038
Median (IQR)	51 (44, 59)	48 (42, 55)	53 (45, 60.5)	
**Intense surveillance**				0.467
No	57 (55.3)	19 (61.3)	38 (52.8)	
Yes	46 (44.7)	12 (38.7)	34 (47.2)	
**Primary cancer character**				
**Histopathology**				0.656
Non-invasive	26 (25.2)	7 (22.6)	19 (26.3)	
Invasive	77 (74.8)	24 (77.4)	53 (73.7)	
Subtype^*^				0.001
Luminal A	26 (26.8)	1 (3.3)	25 (37.3)	
Luminal B	40 (41.2)	15 (50)	25 (37.3)	
HER2^†^	12 (12.4)	3 (10)	9 (13.4)	
TNBC^‡^	19 (19.6)	11 (36.7)	8 (11.9)	
**Staging**				0.096
stage0 & stageI	56 (54.4)	13 (41.9)	43 (59.7)	
stageII & stageIII & stageIV	47 (45.6)	18 (58.1)	29 (40.3)	
**Recurrent cancer character**				0.938
Non-invasive	23 (22.3)	7 (22.6)	16 (22.2)	
Invasive	80 (77.7)	24 (77.4)	56 (77.8)	
Subtype^**^				0.306
Luminal A	13 (13.7)	2 (6.9)	11 (16.7)	
Luminal B	47 (49.5)	13 (44.8)	34 (51.5)	
HER2	13 (13.7)	4 (13.8)	9 (13.6)	
TNBC	22 (23.2)	10 (34.5)	12 (18.2)	

† Human epidermal growth factor receptor 2.

‡ Triple negative breast cancer.

* 6 missing data; The primary cancer subtype was missed in 1 early recurrence group and 5 late recurrence group.

** 8 missing data; The recurrent cancer subtype was missed in 2 early recurrence group and 6 late recurrence group.

### Detectability

Among the patients with suspicious finding in the imaging during postoperative surveillance after BCT, the detectability of pathology-confirmed recurrent lesions in mammography, ultrasound and MRI at the time of recurrence were reviewed. In the early recurrence group, the review of initial breast MRI was conducted to exclude the possibility of missed cancer on initial breast MRI if recurrent lesions were found at other quadrants, contralateral breasts or axilla. As a result, there was no missed cancer. All recurrent cases were detected on breast MRI except for one case. Of the 103 cases, 60 cases (59.4%) were detected on mammography and 71 cases (68.9%) were detected on ultrasound. The number of cases detected on mammography or ultrasound was 84 cases (81.6%). Combined mammography and MRI for detection of recurrent lesion showed a detectability of 100%. There was no significant difference in detectability between early and late recurrence groups in every imaging modality or their combinations.

MRI showed a significantly higher detectability compared to mammography, ultrasound and mammography combined with ultrasound. The detectability of MRI with mammography also showed a significantly higher detectability than others except for MRI alone (Table 3).

**Table 3:** Detectability of recurrent breast cancer by imaging modality.

	Total	Early recurrence	Late recurrence	
	N = 103	N = 31	N = 72	*p* value
Mammography^*^				< 0.001
No	41 (40.6)	13 (43.3)	28 (39.4)	
Yes	60 (59.4)	17 (56.7)	43 (60.6)	
**Ultrasound**				< 0.001
No	32 (31.1)	8 (25.8)	24 (33.3)	
Yes	71 (68.9)	23 (74.2)	48 (66.7)	
**Mammography & Ultrasound**				< 0.001
No	19 (18.5)	7 (22.6)	12 (16.7)	
Yes	84 (81.6)	24 (77.4)	60 (83.3)	
**MRI**				0.158
No	1 (1)	-	1 (1.4)	
Yes	102 (99)	31 (100)	71 (98.6)	
**Mammography & MRI**				Reference
No	-	-	-	
Yes	103 (100)	31 (100)	72 (100)	

* 2 missing data; Mammography was omitted in 1 early recurrence group and 1 late recurrence group.

Of the 102 cases of MR detected cases, 19 cases were detected only on MRI. Seven cases were early recurrence group (22.6%) and 12 cases were late recurrence group (16.7%), which also showed no significant difference between the two groups.

### Imaging analysis

#### Mammography

*Early recurrence group*. Of the 31 early recurrent cases, 17 cases (56.7%) were detected by mammography. Four cases manifested as calcification with suspicious features. Four cases manifested as mass only. There were 6 cases of developing asymmetry, and the other 3 cases were detected as enlarged axillary lymph nodes.

*Late recurrence group*. Of the 72 late recurrent cases, forty-three cases (60.6%) were detected by mammography. Nine cases manifested as masses only, 14 cases as microcalcifications only, and 15 cases as asymmetries only. Three cases were found as mass with microcalcifications, one case as microcalcifications with asymmetries, and one case as asymmetry with enlarged axillary lymph node.

#### Ultrasound

*Early recurrence group*. Of the 31 early recurrent cases, 23 cases (74.2%) were detected on ultrasound. Thirteen (59.1%) cases were shown as mass lesions, which was a significantly larger number than other lesion forms. Other 5 cases were detected as non-mass lesions, 4 cases as enlarged axillary lymph nodes, and one case was depicted as a combination of non-mass lesion and enlarged axillary lymph node.

There was no significant difference between suspicious shape and non-suspicious shape for mass lesions. However, most of the mass lesions were depicted as parallel hypoechoic mass with non-circumscribed margin on ultrasound.

*Late recurrence group*. Of the 72 late recurrent cases, 48 cases (66.7%) were detected on ultrasound. Similar to the early recurrence group, 37 cases (77%) were detected as mass lesions, which was a significantly larger number than other lesion forms. Six cases were depicted as non-mass lesions, 2 cases as calcifications, and 3 cases as enlarged axillary lymph nodes. The mass lesions showed no significantly suspicious morphology and showed parallel hypoechoic features with non-circumscribed margin.

#### MRI

**Fig 2:**
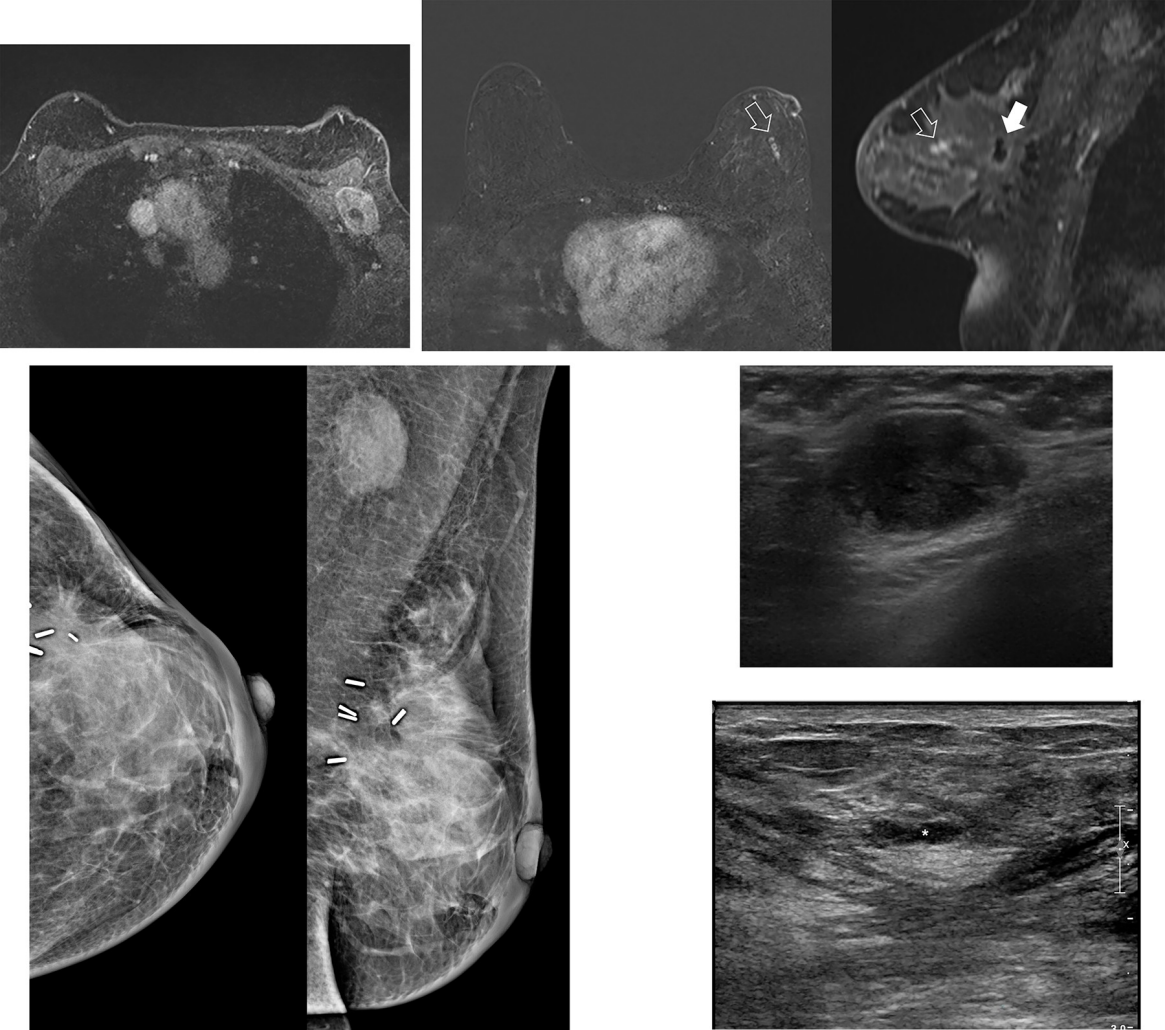
A 40-year-old female patient with an early recurrent breast cancer detected 8 months after BCT. The patient who underwent BCT for left breast cancer (invasive breast cancer, stage II) complained of discomfort in left axilla at the time of postoperative surveillance. Through mammography and ultrasound, an enlarged lymph node was noted. It was confirmed as metastasis by ultrasound-guided core needle biopsy. (a) MRI after confirmed metastatic lymph node in left axilla. A large lymph node, which was confirmed as metastasis, is noted at left axillary level I. (b) At first dynamic phase after contrast enhancement, there is a focal non-mass enhancement (open arrow) at 3 o’clock direction of left breast, anterior to the previous excision site (white arrow). (c), (d) Mammography and ultrasound of the same patient show an enlarged lymph node in left axilla, but there is no significant suspicious finding at excision site on both mammography and ultrasound. (e) On second look ultrasound by breast expert to find non-mass enhancement lesion detected on MRI, an indistinct isoechoic non-mass like lesion (white star) is noted. This lesion was confirmed to be recurrent invasive carcinoma after re-excision with ultrasound-guided needle localization.

*Early recurrence group*. Of the 103 cases, 31 were detected on postoperative breast MRI within 12 months (median 8 months) after BCT. In other words, all early recurrent cases (100%) were detected on postoperative breast MRI. Sixteen breast recurrent cases (51.6%) were found at the same quadrant as the previous lumpectomy site. Six cases were located at the same breast on different quadrant, and the other 4 cases were found at the contralateral breast. Eighteen cases were detected as mass lesions (58.1%), 8 cases as non-mass enhancement (NME), and 5 cases as enlarged ipsilateral axillary lymph nodes (Fig 2).

The mean size of early recurrent lesions was 2.4 cm (±1.7) in the longest diameter. There was no significant difference in morphology between suspicious and non-suspicious features. However, the kinetics of recurrent lesions showed a significant early rapid enhancement pattern (84.1%) regardless of the delayed phase pattern in both mass and NME lesions (Table 4).

**Table 4:** MR characteristics of early and late recurrent lesions.

	Total	Early recurrence	Late recurrence
	N = 103	N = 31	N = 72
**Size**			
Mean ± SD	2.1±1.6	2.4±1.7	1.9±1.5
**Lesion location (n, %)**			
same quadrant with lumpectomy site	52 (50.4)	16 (51.6)	36 (50)
other quadrants with lumpectomy site	18 (17.5)	6 (19.4)	12 (16.7)
contralateral breast	21 (20.4)	4 (12.9)	17 (23.6)
axillary lymph node	12 (11.7)	5 (16.1)	7 (9.7)
**Background parenchymal enhancement (n, %)**			
minimal/mild	87 (84.5)	28 (90.3)	59 (81.9)
moderate/marked	16 (15.5)	3 (9.7)	13 (18.1)
**Form of lesion (n, %)** ^*^			
mass	64 (62.1)	18 (58.1)	46 (63.9)
non-mass enhancement	27 (26.2)	8 (25.8)	19 (26.4)
lymph node	12 (11.7)	5 (16.1)	7 (9.7)
**Feature of mass lesions (n, %)**			
**Shape**			
non-suspicious	28 (43.9)	9 (50)	19 (41.3)
suspicious	36 (56.1)	9 (50)	27 (58.7)
*p* value	0.325	>0.999	0.097
**margin**			
circumscribed	30 (46.9)	9 (50)	21 (45.7)
non-circumscribed	34 (53.1)	9 (50)	25 (54.3)
*p* value	0.484	>0.999	0.412
**enhancement pattern**			
homogenous	27 (42.2)	7 (38.9)	20 (43.5)
non-homogenous	37 (57.8)	11 (61.1)	26 (56.5)
*p* value	0.079	0.189	0.215
**Kinetics** ^**^			
**Early phase**			
rapid	57 (90.5)	14 (82.4)	43 (93.5)
non-rapid	6 (9.5)	3 (17.7)	3 (6.5)
*p* value	< 0.0001	0.0002	< 0.001
**Late phase**			
washout	42 (66.7)	8 (47.1)	34 (73.9)
non-washout	21 (33.3)	9 (52.9)	12 (26.7)
*p* value	0.0002	0.739	< 0.001
**Feature of non-mass lesions (n, %)**			
**distribution**			
non-segmental	19 (73.1)	5 (62.5)	14 (77.8)
segmental	7 (26.9)	3 (37.5)	4 (22.2)
*p* value	0.001	0.48	0.001
**enhancement**			
homogenous	3 (11.4)	-	4 (22.2)
non-homogenous	23 (88.5)	8 (100)	14 (77.8)
*p* value	< 0.001	-	0.001
**Kinetics**			
**Early**			
non-rapid	5 (19.2)	-	5 (27.8)
rapid	21 (80.8)	8 (100)	13 (72.2)
*p* value	0.002	-	0.059
**Late**			
non-washout	11 (42.3)	4 (50)	7 (38.9)
washout	15 (57.7)	4 (50)	11 (61.1)
*p* value	0.433	> 0.999	0.346

* 1 missing data; One case that was not detected in MRI was missing because it was impossible to analyze lesion characteristics.

** 1 missing data; The kinetics analysis was missing in one early recurrence group.

**Fig 3:**
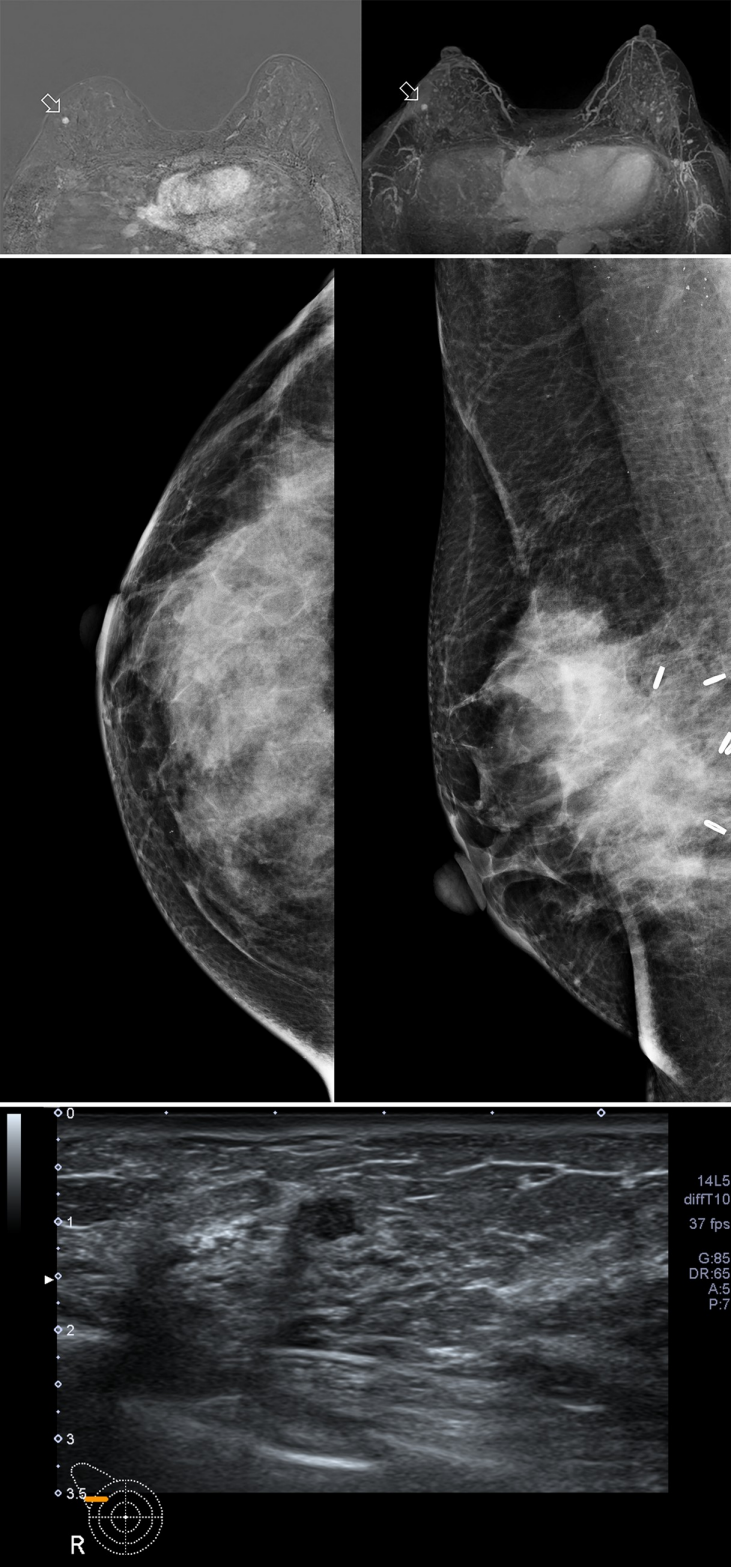
A 43-year-old female patient with a late recurrent breast cancer detected 20 months after BCT. (a) MRI of a 43-year-old patient who underwent BCT for right breast cancer (invasive ductal carcinoma, stage II). The patient underwent BCT alone and refused radiation therapy and chemotherapy after surgery. On the second annual postoperative MR surveillance, a small (0.6cm) sized enhancing nodule is detected at the upper outer quadrant of right breast (10 o’clock direction, open arrow). (b), (c) Mammography and ultrasound of the same patient for routine postoperative surveillance. There is no suspicious finding on mammography. On the second look ultrasound, there is a small hypoechoic nodule at the possible concordant location with the MR detected lesion. This lesion was underestimated before MRI examination due to multiple hypoechoic nodules at upper outer quadrant of right breast. It was confirmed as recurrent invasive ductal carcinoma by core needle biopsy.

Of the 31 early recurrent cases, 7 cases were detected on MRI only. Most of those lesions (6 of 7) were 1 cm or larger (mean size 1.96 cm [± 0.78]) and were detected in the same quadrant as the previous lumpectomy site (5 of 7) (Fig 3).

**Fig 4:**
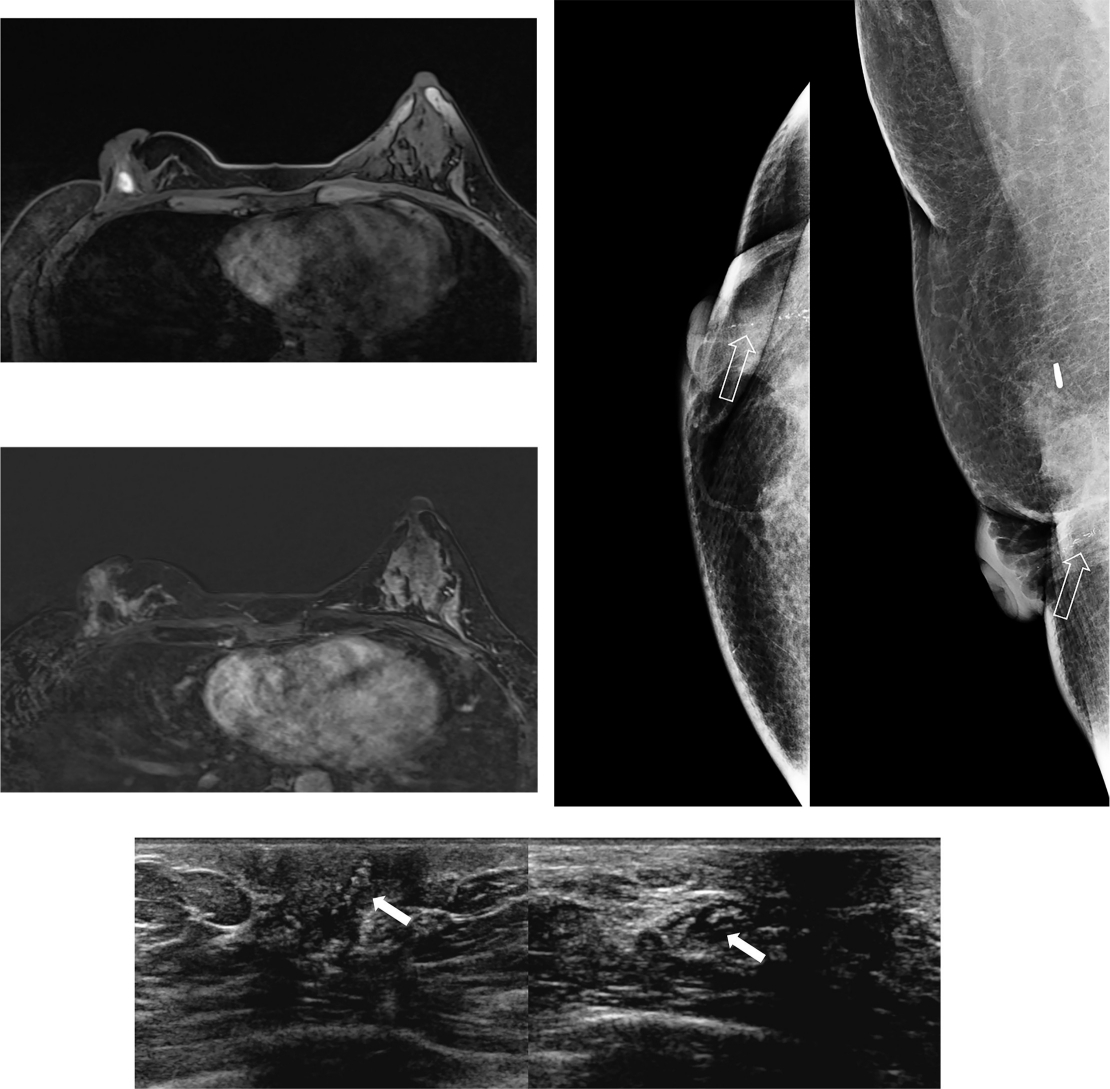
A 35-year-old female patient with a late recurrent breast cancer detected 30 months after BCT. (a), (b) MRI after recurrent ductal carcinoma in situ of right breast was confirmed through ultrasound-guided biopsy. Only hematoma is detected at right subareolar region on pre-contrast fat saturated T1WI (a), and no definite enhancement is noted on subtracted early dynamic phase of contrast enhanced fat saturated T1WI (b). (c) Mammography of the same patient for routine postoperative surveillance. Mammography showed linearly distributed fine pleomorphic microcalcifications (open arrow) at right subareolar region, adjacent to previous BCT site. (d) Ultrasound of the same patient for routine postoperative surveillance. Ultrasound showed intraductal echogenic foci (white arrow) at right subareolar region and nipple.

*Late recurrence group*. Of the 72 late recurrent cases, 71 cases (98.6%) were detected on postoperative breast MRI. The only case not detected on MRI was recurrent ductal carcinoma in situ case which was manifested as microcalcification on postoperative mammography. It showed no enhancing lesion on MRI after biopsy (Fig 4).

**Fig 5:**
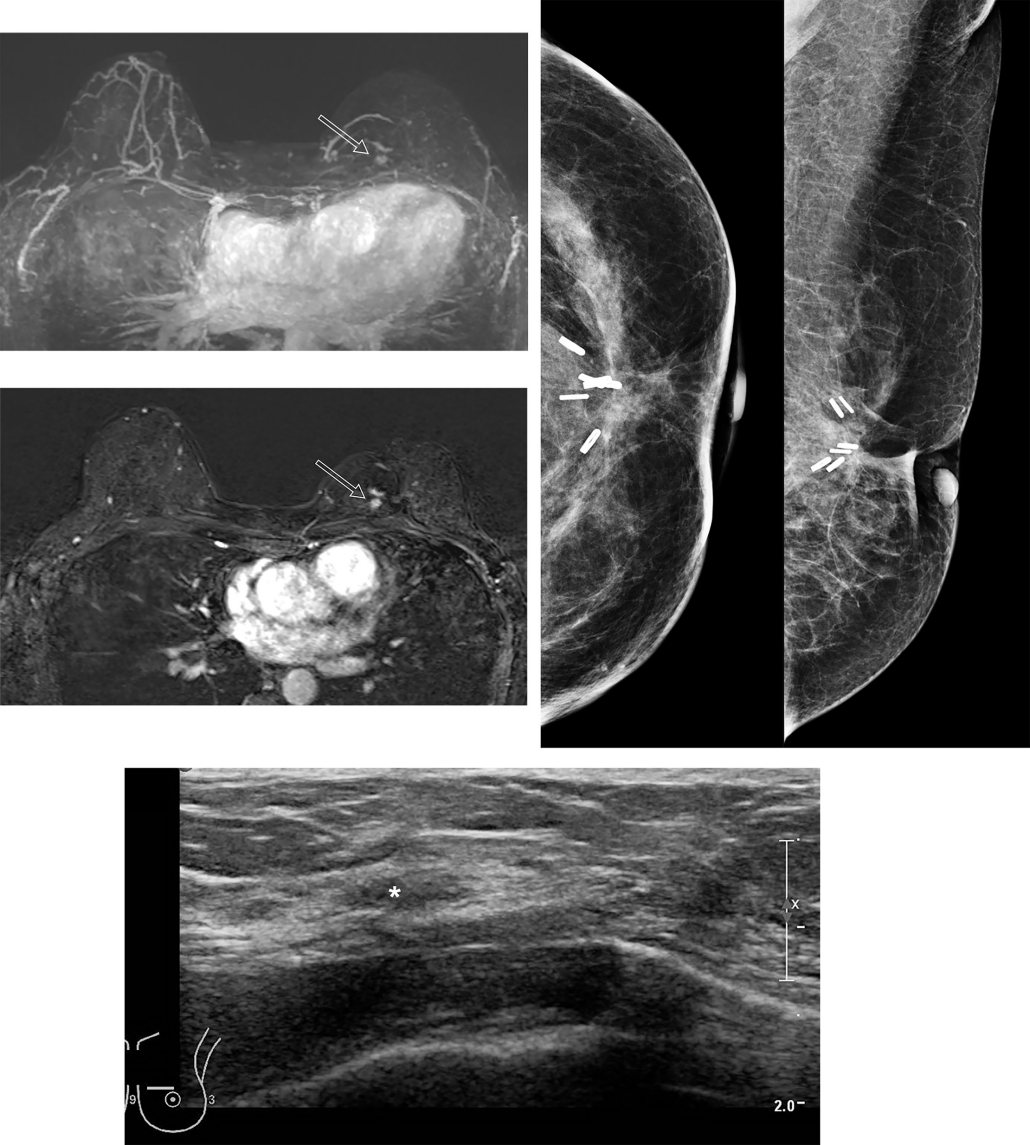
A 62-year-old female patient with an early recurrent breast cancer detected 12 months after BCT. (a) MIP image of postoperative MRI after 12 months from BCT for left breast cancer. There is a prominent nodular enhancement at upper inner quadrant of left breast (open arrow). (b) On subtracted early dynamic phase of contrast enhanced T1WI, a brightly enhancing nodule is noted adjacent to previous BCT site (open arrow). (c) There is no abnormal finding on mammography of the same day as breast MRI. (d) Second look ultrasound for biopsy shows oval parallel, indistinct isoechoic lesion (white star) mimicking normal parenchymal pattern at corresponding site of enhancing lesion detected on MRI. This lesion was not detected on breast ultrasound performed at the same day as breast MRI.

Thirty-six of 72 cases (50%) were detected at the same quadrant as the lumpectomy site. Larger number of late recurrence group (17 cases, 24.6%) was detected at the contralateral breast compared to the early recurrence group (12.9%) without significant difference (Fig 5).

Forty-six cases (66.7%) were mass lesions, 19 cases manifested as NME and 7 cases as enlarged ipsilateral axillary lymph nodes in the late recurrence group.

The morphology of mass lesions in late recurrent group also showed no significant difference between suspicious and non-suspicious features, which was similar to the early recurrent lesions. However, recurrent lesions showed non-segmental distribution or non-homogenous enhancement pattern more than segmental distribution or homogenous enhancement pattern in cases of NME. The recurrent lesions of the late recurrence group showed rapid enhancement in early phase as did the lesions of the early recurrence group. However, more than half of the late recurrent lesions (34 masses, 11 non-mass, 60.8%) showed a washout pattern with significance unlike the early recurrence group (Table 4).

Of 72 cases of late recurrence, 12 cases were only detected on MRI. The mean size of those lesions was 1.35 cm (± 0.37). Five of those cases were detected in the same quadrant as the previous lumpectomy site, and the other cases were detected in other quadrant from lumpectomy site (4 cases), in contralateral breast (2 cases), or as lymph node of ipsilateral internal mammary chain with previous BCT side.

## Discussion

Contrast enhanced breast MRI is the most sensitive imaging method for detecting breast malignancy. In particular, the detection rate of recurrent or residual tumors after BCT was reported to be over 90% in previous studies [12–15]. However, NCCN does not recommend breast MRI as a routine postoperative surveillance for women with history of breast cancer [3, 16] due to insufficient evidence and its high cost [8, 17, 18]. Annual mammogram alone is recommended as the postoperative surveillance by NCCN guideline. However, American college of Radiology recently recommended that contrast enhanced breast MRI should be considered in patients with personal history of breast cancer. This is because contrast enhanced breast MRI shows fewer false positives, higher specificity, and equivalent sensitivity and cancer detection rate in patients with history of breast cancer compared to other women with high risks such as genetic or family history [19]. The results of our study also suggest that MRI shows a significantly higher detectability of 99% for recurrent cancer in both early and late recurrence groups whereas mammography shows a detectability of 59.4% for recurrent lesions.

In post-BCT breasts, enhancement from non-specific postoperative change or healing process is frequently detected and these findings are frequently confused with residual or recurrent tumor [20, 21]. However, we identified the kinetic characteristics of recurrent lesions and the detection rate of MRI. Recurrent lesions detected on postoperative breast MRI showed a fast enhancement in early phase in both early and late recurrence groups. In early recurrence group, kinetics of delayed phase was variable whereas washout pattern was significantly larger in number than other patterns in late recurrent group. In other words, any fast-enhancing lesion in early phase on postoperative MRI within 12 months after BCT should be emphasized regardless of its delayed kinetic pattern; and further workup such as ultrasound-guided biopsy or MR-guided biopsy should be recommended. These results could also be supported by the fact that non-specific enhancement associated with postoperative changes shows slow initial enhancement with persistent delayed enhancement in a previous study [22].

The mean size of early recurrent lesions (2.4 cm) had a tendency to be larger than that of late recurrent lesions (1.9 cm, *p* = 0.06). It could be explained by the aggressiveness of early recurrent tumors such as triple negative subtype. In fact, the number of triple negative subtype was slightly larger in the early recurrence group (34.5%) than in the late recurrence group (18.2%) (*p* = 0.07).

The median interval between BCT and recurrence was 8 months in early recurrence group and half of the recurrent lesions were detected at the same quadrant as the lumpectomy site. Post-surgical changes and radiation therapy related changes could be severe, especially at the lumpectomy site, masking recurrent lesions on mammography and ultrasound within 6 to 12 months after BCT [23–25]. In our study, early recurrent lesions developed not only as mass lesions, but also as non-mass lesions or axillary lymph nodes. These may be limited in detection on mammography or ultrasound because of postoperative changes. In imaging analysis, mammography had limitations in detecting recurrence due to postoperative changes, especially when in dense breast. Ultrasound to compensate for limitation of mammography also had limitations. Although it had less limitations than mammography in detecting lesions, it is possible that small recurrent lesions or non-mass lesions may have been missed. Such lesions may have been missed especially when ultrasound was performed by less experienced radiologists because we only reviewed captured images, not the real-time images. However, postoperative MRI could detect recurrent lesions more easily without diagnostic delay of recurrence.

Nineteen cases of early and late recurrence groups were detected only on MRI. Of the seven cases of early recurrence group, 6 lesions were developed at the same quadrant as the lumpectomy site. The size of these cases was larger than 1 cm at the time of detection. Of the twelve cases of late recurrence group, half of the lesions were developed at a different quadrant from the lumpectomy site. The number of lesions which measured 1 cm or larger was smaller than that of the early recurrence group. In other words, the MR-only detected late recurrent lesions had a tendency to be smaller than early recurrent lesions. Also, they tended to develop at an unexpected site including different quadrant from the lumpectomy site. However, there was no statistically significant difference between the two groups due to the small number of cases. This finding suggests that breast MRI can be helpful in detecting disease recurrence at least 12 months after BCT. If the lesions are smaller than 1cm or located at an unexpected site of breast, the lesions could be missed or be underestimated on mammography or ultrasound.

There are a few limitations in our study. First, this is a retrospective study from a single institution. The small number of cases in both early and late recurrence groups and the different time interval between first postoperative MRI and BCT for each patient could be considered as a limitation. In addition, there was no case of MR-guided biopsy in recurrent cases, especially for the cases detected only on MRI. Despite the MR only detected cases, the absence of a case diagnosed with MR-guided biopsy can also be a limitation. However, MR-guided biopsy was very limited due to the condition of breast after BCT or other conditions such as small breast volume or location of enhancing lesions. For these reasons, second-look ultrasound was performed by breast expert radiologists for ultrasound-guided biopsy or preoperative localization. Most of the lesions showed benign-mimicking features on ultrasound that were difficult for detection even by experts. However, they were easily detected on MRI due to contrast enhancement. This fact may also support the usefulness of postoperative breast MRI.

In summary, early fast enhancement is the most important feature of recurrent lesions on postoperative breast MRI for both early and late recurrence groups. Any new lesion with size of 1cm or larger detected on MRI within 12 months after BCT at the same quadrant as the lumpectomy site, should be considered suspicious and further workup is needed. Also, if any new lesion shows early fast enhancement and delayed washout pattern regardless of its location and size after 12 months from BCT, emphasis should be made on the lesion due to the possibility of recurrence.

## Conclusion

Postoperative breast MRI shows very high detection rate for recurrent breast cancer including recurrent cancers that were not found on mammography or ultrasound. In addition to its high detectability, characteristic features of recurrent lesions are noted on MRI according to the time of recurrence. These results are not only the milestone for proving the usefulness of the postoperative breast MRI, but also the basis for determining the appropriate time interval for postoperative breast MRI after BCT.

## Supporting information

S1 Raw data (XLSX)
